# Choline‐Based Deep Eutectic Solvents for Enzymatic Preparation of Epoxy Linseed Oil

**DOI:** 10.1002/elsc.70016

**Published:** 2025-03-17

**Authors:** Hui Zhang, Kai Wang, Shuai Huang, Ziheng Cui, Biqiang Chen

**Affiliations:** ^1^ College of Life Science and Technology Beijing University of Chemical Technology Beijing P.R. China

**Keywords:** chemo‐enzymatic epoxidation, deep eutectic solvents, linseed oil, ‘self’‐epoxidation

## Abstract

Deep eutectic solvents (DESs) hold the potential to serve as a sustainable and environmentally friendly substitute for supercritical fluids, ionic liquids, and organic solvents. Moreover, DESs have been demonstrated to assist in stabilizing the structure of enzyme. The enzymatic synthesis of epoxy vegetable oil in a DES‐system was developed in this study, and the influence of DESs viscosity on the epoxidation system was investigated for the first time. The results demonstrated that the epoxy value reached 8.97, and the double bond conversion rate was 82.48%. The viscosity of the reaction system decreased from 209.32 to 91.35 (mPa·s). The application of DES in epoxidation was confirmed through structural characterization, indicating that eutectic solvents could serve as substitutes for toxic and volatile organic solvents in synthesizing high‐epoxide vegetable oils using an enzymatic method, thus facilitating the production of environmentally friendly plasticizers.

## 1 Introduction

Epoxy vegetable oil is a biobased product formed by the epoxidation of vegetable oil using hydrogen peroxide (H_2_O_2_). It has advantages such as low cost, low toxicity, renewability, and high chemical reactivity. It can be used as a plasticizer [1, 2, 3, 4], and stabilizers [5] for various polymers (chlorinated rubber, polyvinyl chloride (PVC), and polylactic acid and so forth [6, 7, 8], as well as for monomer synthesis and polymer preparation of epoxy resins, making it a crucial industrial chemical.

Currently, the main process for oil epoxidation is still the traditional Prileschajew chemical catalysis method based on in situ formation of percarboxylic acid and transfers activated oxygen to unsaturated compounds, which involves the use of excessive H_2_O_2,_ strong inorganic acids, and organic acids (such as formic acid and acetic acid) as chemical catalysts [9, 10]. This leads to equipment corrosion, the generation of large amounts of wastewater, and the difficult‐to‐recover by‐products, resulting in deficiencies in process greening and safety. Therefore, finding efficient biocatalysts and developing mild and environmentally friendly biocatalytic techniques has always been a focus of attention for the industry and researchers [11, 12].

The chemo‐enzymatic epoxidation is an alternative method [13], using enzymes as biocatalysts to generate percarboxylic acid instead of strong acidic catalysts [14, 15, 16]. Vegetable oils from Soybean [17], sunflower, and linseed [18] were epoxidized in the presence of hydrophobic organic solvents [19], such as toluene [20], with a conversion above 80%. However, the addition of organic solvents had certain harm to the environment and organisms, which was contrary to the current concept of “greenness”. Several attempts have been made to solve these issues, such as using other green solvents like ionic liquids [21, 22] and supercritical fluid [23]. However, their high costs, poor biodegradability, poor biocompatibility, and low sustainability greatly limit their technical applicability [24]. Therefore, there is an urgent need to seek a lower cost ideal sustainability solvent that has high substrate solubility, high enzyme activity and stability, positive impact on reaction equilibrium.

As a more promising application technology, DESs offer various advantages, including low cost, high availability, low toxicity, high biodegradability, and reproducibility [25, 26, 27, 28]. A growing number of studies of DESs as environmentally acceptable or better alternatives to established reaction solvents in biotransformation. DESs are usually prepared by mixing a hydrogen bond acceptor (HBA)‐quaternary ammonium salts and a hydrogen bond donor (HBD)‐alcohols, amides, carboxylic acids, and polyols. Currently, deep eutectic solvents (DESs) have found extensive application in the synthesis of various materials, including covalent organic frameworks (COFs) [29, 30], nanomaterials [31], and ionic gels [32] and it had been also used in biocatalysis, such as esterification [33], transesterification [34, 35], and amidation [36]. Many DESs are enzyme‐friendly [37, 38, 39], including the use of hydrolases [40, 41], lyases [42], oxidoreductases [43], and even whole cells [44]. The formation of intermolecular hydrogen bonds leads to a decreased freezing point, and the DES remains a liquid at ambient temperature [33, 45]. In recent years, Wu et al. present an enzyme‐friendly natural deep eutectic solvent (NADES)‐based medium for the one‐pot chemobiocatalytic valorization of fructose into valuable furanics via five‐hydroxymethylfurfural (HMF). The NADES‐based medium was benign toward various biocatalysts such as alcohol and aldehyde dehydrogenases, acyltransferase, and ω‐transaminase, evidenced by good yields (86%−97%) of the desired products in subsequent biotransformations [38]. In 2023, Sobhan et al. successfully used DESs based on choline chloride–oxalic acid (DES‐02) and choline chloride–butyric acid (DES‐06) to catalyze the epoxidation of soybean oil, the results showed an optimal conversion with a high selectivity of 73% when soybean oil was epoxidized with bifunctional DES‐02 catalysts [46]. The use of DESs has great potential to address the problems of using organic solvents in the current biocatalysis process for epoxidation of vegetable oil.

**SCHEME 1:**
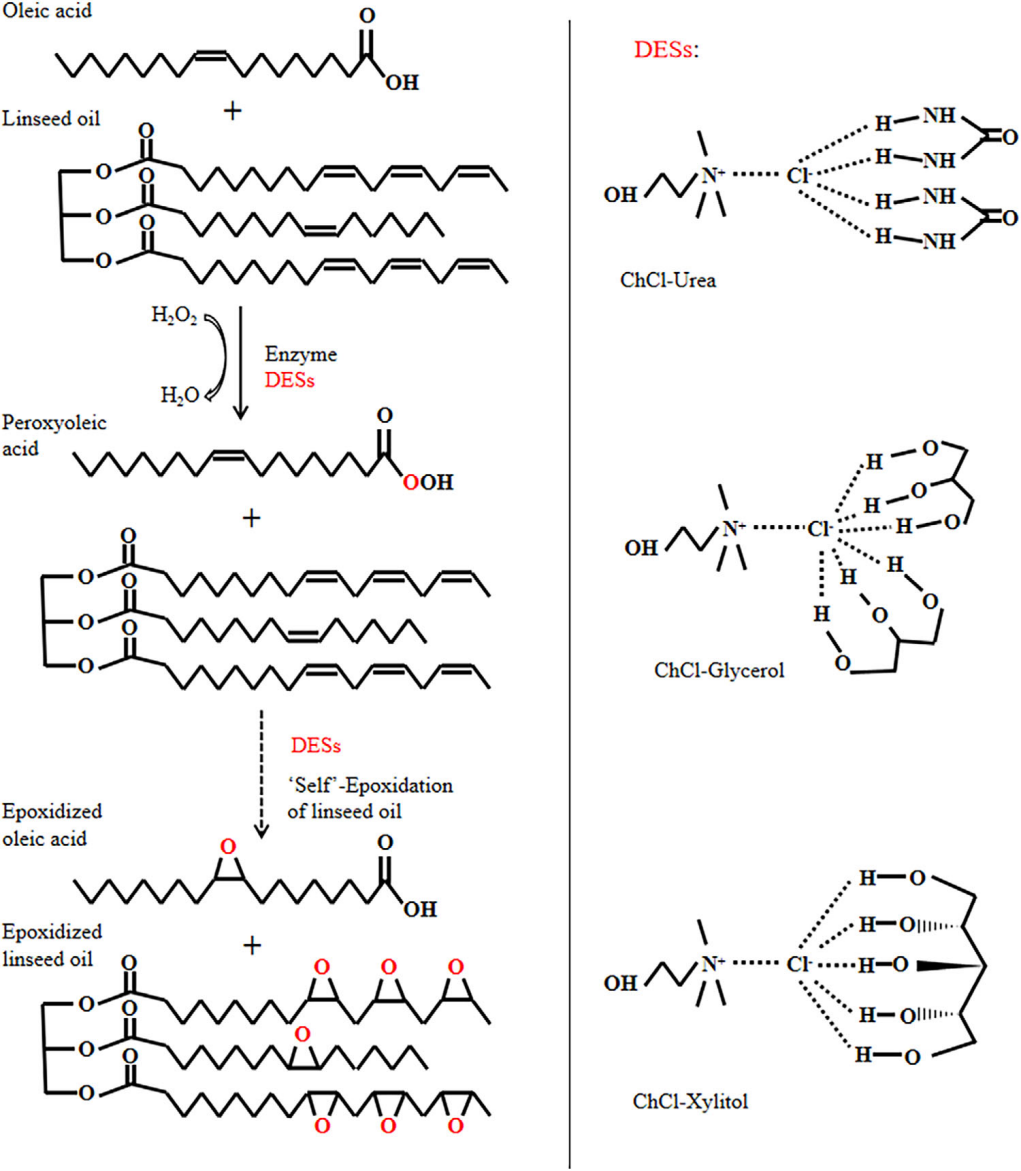
Chemo‐enzymatic ELO with addition of oleic acid to the reaction mixture; DESs added as solvent.

In this study, the utilization of DES for enzymatic synthesis of epoxy vegetable oil was successfully developed, and the impact of DES viscosity on reaction mass transfer was discussed for the first time. The epoxy oil with a high epoxide value can be obtained while maintaining low viscosity and high mass transfer, with an epoxide value exceeding 8.5. In terms of usage, environmental protection, and economic aspects, it is superior to toxic and harmful volatile organic solvents. Linseed oil containing more linolenic acid with three unsaturated double bonds was used as the reaction raw material for epoxidation, and its potential applications in plastics manufacturing were investigated [47]. The epoxidation of vegetable oils was catalyzed by an enzyme, and “self”‐epoxidation of vegetable oil was achieved by adding oleic acid (Scheme 1). The effects of DESs proportion, reaction temperature, and viscosity on epoxidation were discussed and target compounds were characterized using ^1^H NMR, ^13^C NMR, Fourier transform infrared (FT‐IR), and thermogravimetric analysis (TGA).

## 2 Materials and Methods

### 2.1 Materials

Linseed oil (iodine value of 173.1 g of I2/100 g) was acquired from Shandong Jiqing Chemical Co., Ltd. (Shandong Province, China). Chemical reagents used in the experiment included choline chloride (AR, 98.5%), xylitol (AR, 98.5%), glycerol (AR, 99%), urea (AR, 99%), D‐sorbitol (AR, 99%), ethylene glycol (AR, 99%), α‐D‐glucose (AR, 99%), urea hydrogen peroxide (AR, 99%), sodium hydroxide (AR, 99%), anhydrous sodium sulfate (AR, 99%), hydrogen peroxide (AR, 30%), hydrochloric acid (AR, 99%), acetone (AR, 99%), n‐hexane (AR, 99%), and methanol (AR, 99%). The epoxidation was carried out in a parallel synthesis reactor, and the product was analyzed by KerAV‐600 MHz spectrograph and Nicolet Tensor 270 spectrograph (Bruker). The viscosity of the DES was determined by TA Discovery HR‐2 Hybrid Rheometer. The boron trifluoride‐methanol solution (14% in methanol), and sodium chloride (AR, 99%) were also used in the experiment.

### 2.2 Screening of the DESs

Previous experience had shown that a temperature range of 30°C–50°C is optimal for lipase‐catalyzed reactions. However, during the synthesis process, DESs form a hydrogen‐bond network, resulting in a solution with a certain viscosity. The viscosity of the DES varies depending on the solvent used. In this study, DESs that maintain a flowing state at 50°C were chosen for the reaction system to avoid negative impacts on mass transfer. Common DESs used in lipase‐catalyzed reactions were identified and used as solvents for the epoxidation of vegetable oil. Choline chloride served as the hydrogen‐bonding receptor, and alcohols, amides, sugars, and sugar alcohols served as hydrogen‐bonding donors to synthesize DESs, which were added to the epoxidation reaction as solvents. The method of solvent preparation is elaborated in the Supplementary Materials.

### 2.3 Epoxidation

In a round bottom flask equipped with a parallel synthesizer, 25 g of linseed oil, 1.25 g (5% w/w) of oleic acid, and 25 g of DESs (with a ratio of 1:1 to linseed oil) were mixed. Slowly, 19 mL of 30% hydrogen peroxide was added dropwise through a syringe infusion pump set to a speed of 1 mL/h, to achieve a 1:1 molar ratio based on the number of double C═C bonds in the fatty acids. Then, 2.5 g of Novozym435 enzyme was added to the reaction medium. The mixture was stirred at 400 rpm and heated to the designated temperature for 20 h. Samples were withdrawn every 2 h and centrifuged for 3 min at 4500 rpm, with the oil phase containing the target product and the aqueous phase containing the lipase and DESs. The organic phase was washed with warm distilled water (50°C), and the remaining oil was obtained as epoxidized linseed oil after removing low‐boiling point impurities using a rotary evaporator. The aqueous phase containing DESs was vacuum distilled to remove excess water and recover the DESs. The double bond conversion rate and the calculation method of epoxy value are presented in the supplementary document.

## 3 Results and Discussion

### 3.1 Characterization of DESs

**FIGURE 1:**
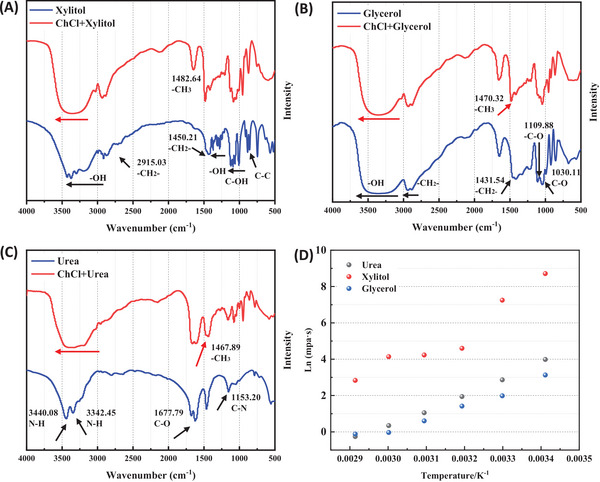
FT‐IR spectra of (A) xylitol and ChCl–xylitol (B) urea and ChCl‐urea (C) glycerol and ChCl‐glycerol. The groups associated with wave numbers highlighted by the arrows. (D) Plot of the logarithm of viscosity versus the reciprocal of temperature for DESs.

To account for the structural differences between the DESs, the FT‐IR spectra were detected individually for each compound. In addition, the FT‐IR spectra of the individual components of the DESs (xylitol, urea, and glycerol) were also analyzed, and the results are presented in Figure 1A–C. The absence of a methylene group in the urea structure resulted in the absence of a stretching vibration of ─CH_2_─ at 3000–2700 cm^−1^. The peaks observed at 3342.45 and 3440.08 cm^−1^ in the FT‐IR spectra of urea were due to the stretching vibration of N─H. The peaks of ─C═O at 1677.79 cm^−1^ and the peaks of C─N at 1153.20 cm^−1^ in the urea structure were also observed (Figure 1B). In the spectra of xylitol and glycerol (Figure 1A,C), the peak of ─OH stretching vibration was observed at 3200–3500 cm^−1^. The peaks at 1300–1500 cm^−1^ were attributed to the bending vibration of ─OH, and the strong stretching vibration of C─OH at 1025–1200 cm^−1^ indicated the presence of alcohols. The peak of C─OH in xylitol showed stronger stretching vibration than that in the peak of glycerol. The peaks at 2915.03 cm^−1^ in xylitol and 2937.67 cm^−1^ in glycerol were due to symmetrical stretching vibration of the methylene groups. The spectra of xylitol and glycerol at 1450.21 and 1431.54 cm^−1^ showed the bending vibration of methylene group. The peak between 890 and 850 cm^−1^ indicated the existence of C─C bonds.

In addition, Figure 1 shows the FT‐IR spectra of the different DESs. The stretching vibration absorption bands of ─OH in urea, xylitol, and glycerol exhibited a red shift and became broader, presumably due to the formation of a large number of hydrogen bonds during the synthesis of the DESs. The enhanced viscosity resulted from the high molecular weight and more hydrogen bonding in stronger van der Waals interactions, which led to an increase in viscosity. The C─H stretching vibrations at 2915.03 cm^−1^ in xylitol and 2937.67 cm^−1^ in glycerol shifted to 2940.12 and 2950.12 cm^−1^, respectively. While the peak of ─C═O in urea exhibited the same shift. Due to the addition of ChCl, the wagging vibration of the terminal methyl in ChCl was also observed in these three DESs. They were located at 1482.64, 1470.32, and 1467.89 cm^−1^ in ChCl−xylitol, ChCl−glycerol, and ChCl−urea, respectively. This indicated that the molecular structure of ChCl remained unchanged during the synthesis of the DESs. The DESs were also analyzed by ^1^H NMR and ^13^C NMR simultaneously, and the corresponding spectra were shown in Figures S3–S8.

A complex hydrogen bonding network exists between HBD and chloride ions in DESs. As a result, DESs exhibit higher viscosity, during epoxidation the viscosity played a significant role in mass transfer. The viscosity affects both hydrogen bonding and mass transfer. Figure 1D illustrates the correlation between the logarithm of viscosity and the reciprocal of temperature for three DESs. The viscosity changes considerably with temperature, decreasing as the temperature increases [48]. This can be attributed to hydrogen bonds being weakened at higher temperatures. The figure shows that ChCl−xylitol has the highest viscosity of the three DESs, reaching 70.53 mPa·s at 70°C. Conversely, ChCl−glycerol and ChCl−urea have lower viscosities, which is theoretically beneficial for mass transfer in the epoxidation system, at only 53.98 and 53.21 mPa·s at 70°C, respectively.

### 3.2 Screening of the DESs

**FIGURE 2:**
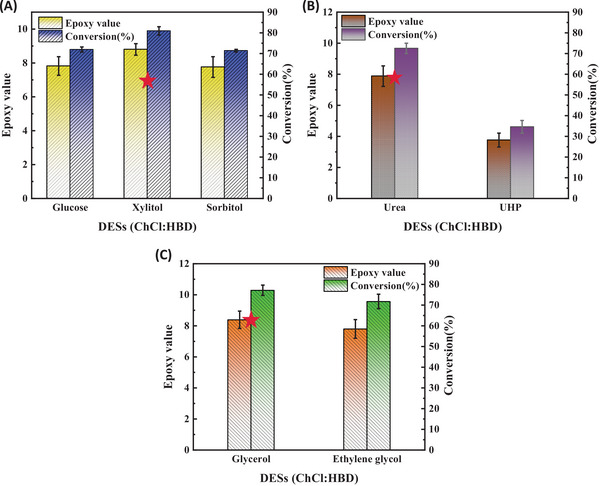
ELO with different DESs. (A) Sugar alcohols; (B) amides; (C) alcohols.

Based on the preliminary screening results from Experiment 2.2, seven types of DESs that retain fluidity at 50°C were selected for the enzymatic epoxidation of vegetable oil. By utilizing DESs as solvents for ELO, it was found that DESs offered several advantages over organic solvents. Through a comparison of the epoxy value and conversion of epoxidation with different solvents, it was determined that DESs were not only environmentally friendly, but also had many application advantages. DESs were easy to handle due to their characteristic of being easily soluble in water, and the conversion could reach over 70% (as shown in Table 1). Simultaneously, to further demonstrate the advantages of DESs in enhancing enzyme stability, we conducted a comparative analysis of enzymatic synthesis of epoxy vegetable oil using three different reaction systems: with DES, without any solvent, and with an organic solvent. As illustrated in Figure S9, the reaction system devoid of any solvent exhibited a significantly diminished epoxidation efficiency due to hydrogen peroxide‐induced damage to the biocatalyst, resulting in an epoxidation value of merely 6.67. In contrast, both the DES and organic solvent systems achieved epoxidation values exceeding 8. Given its environmental benefits, DES was selected for further investigation. This comparison underscores the critical role of DES in enhancing the enzymatic synthesis of epoxy vegetable oil and highlights the enzyme's limited resistance to H_2_O_2_. This study significantly improved lipase tolerance to H_2_O_2_ by leveraging the stabilizing effect of DESs on biological enzymes. Additionally, DESs were environmentally friendly and the product was easy to separate, and DESs could be reused. The conversion and epoxy value of the epoxidation using three different DESs, including the xylitol system, urea system, and glycerol system, were found to be 79.76%, 72.49%, and 77.18%, respectively, as shown in Figure 2A–C. Therefore, these three DES systems were selected for further study.

**TABLE 1:** The DESs were added to the epoxidation reaction as solvents.

No.	HBD	Type	ChCl:HBD	T (°C)	Conversion (%)	Fluidity at 50°C
**1**	Glycerol	Alcohol	1:2	−40	77.18	YES
**2**	Ethylene glycol	Alcohol	1:2	−20	71.76	YES
**3**	Urea	Amide	1:2	12	72.49	YES^a^
**4**	Urea hydrogen peroxide (UHP)	Amide	1:2	/	31.92	YES
**5**	D‐Glucose	Sugar	5:2	14	72.31	YES
**6**	D‐Sorbitol	Sugar Alcohol	1:1	RT	72.31	YES
**7**	D‐Xylitol	Sugar Alcohol	1:1	RT	79.76	YES

^a^ Liquid, but highly viscous. *T*°C referred to the melting point of the mixtures. RT referred to room temperature. Conversion referred to the amount of plants oil converted to its respective epoxide.

### 3.3 Effects of Different Factors on the Reaction

In the enzymatic epoxidation of vegetable oils, we not only chose linseed oil, which has a high content of unsaturated fatty acids, but also employed a flow addition method to introduce an excess of hydrogen peroxide. This approach helps prevent the decomposition of hydrogen peroxide and also provides a buffering effect to mitigate its potential damage to the enzyme. Moreover, regarding enzymatic epoxidation of vegetable oils, a study as early as 1999 proposed that, with a small addition of unsaturated fatty acids, lipase could catalyze the “self”‐epoxidation of vegetable oils, leading to the formation of primarily epoxidized triglycerides and a small amount of epoxidized free unsaturated fatty acids [49]. Building upon these foundational studies, we are further optimizing the epoxidation system using DESs.

**FIGURE 3:**
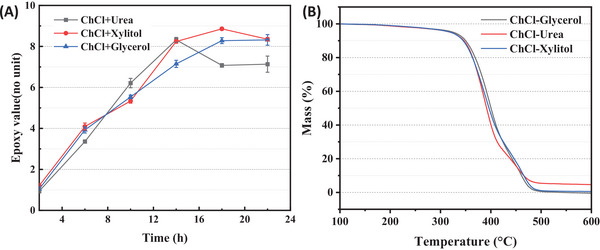
(A) ELO with DESs; (B) TG curves of ELO with DESs.

Reaction Time: The lipase‐catalyzed in situ formation of peroxy fatty acids is a highly selective method for the epoxidation of plant oils [49]. However, the enzyme's H_2_O_2_‐resistance is poor. The tolerance of the lipase to H_2_O_2_ was reinforced in this study through the stabilization effect of DESs. Three DESs were selected based on their viscosity, and their efficacy was tested in epoxidation reactions conducted for different reaction times. The results of epoxidation in the three DESs were shown in Figure 3A.

It was observed that the epoxy value of the epoxidation improved over time. As the reaction time varied from 2 to 22 h, the epoxy value in all three DESs gradually increased (1.5–8.5). ChCl−urea had the highest epoxy value of 8.3 for 14 h. In contrast, it took 18 h and 22 h to achieve the highest epoxy value of 8.8 for ChCl−xylitol and 8.6 for ChCl−glycerol, respectively. Moreover, the epoxy value had started to decline to varying degrees after 14 h for ChCl−urea and after 18 h for ChCl−xylitol. This indicated that the phenomenon of side reaction was severe when using ChCl−urea as a solvent, and the epoxy value sharply decreased from 8.3 to 7.1 within 4 h.

The highest epoxy value of the ChCl−xylitol and ChCl−glycerol systems was 8.9 and 8.4, respectively. In the ChCl−xylitol system, the epoxy value slightly decreased to 8.3. However, in the ChCl−glycerol system, the epoxy value never decreased. This demonstrated that the epoxy value of epoxidation in ChCl−urea increased more slowly, although the reaction rate was fast, which could be attributed to less HBD. However, the ChCl−xylitol and ChCl−glycerol systems provided more HBD and enhanced the hydrogen bonding force, contributing to the progress at the conversion of epoxidation.

Regarding conversion, it was observed that there was an increase with reaction time, but a narrow difference existed after 14 h. In the ChCl−urea system, the conversion of double bonds for 14 h was 76.3%, but the reaction rate decreased rapidly after 14 h, which was probably significantly affected by the concentration of hydrogen peroxide. In the ChCl−xylitol and ChCl−glycerol systems, the highest conversion of double bonds was 81.8% and 77.2%, respectively. In principle, the concentration of hydrogen peroxide would decrease with reaction time.

Reaction Temperature: To increase the efficiency of epoxidation based on DESs, we conducted the ELO at different temperatures (30°C–60°C) for 18 h. Table 2 presents the conversion, epoxy value, and viscosity results for three DESs at different reaction temperatures. The highest conversions of double bonds were achieved at 60°C with ChCl−xylitol (82.5%), ChCl−glycerol (77.8%), and ChCl−urea (76.8%). The corresponding epoxy values were 8.97, 8.46, and 8.35, respectively.

**TABLE 2:** List of three DESs used as solvents for epoxidation at different temperature.

	ChCl─xylitol			ChCl─urea			ChCl−glycerol		
*T*°C	EV^a^	C^b^.%	V^c^.mPa·s	EV	C.%	V.mPa·s	EV	C.%	V.mPa·s
30	7.05	64.82	645.78	7.21	66.29	526.43	7.81	71.82	217.78
40	7.21	66.29	390.12	7.53	69.24	279.16	8.23	75.68	164.95
50	8.83	81.19	209.32	8.30	76.32	143.12	8.37	76.97	91.35
60	8.97	82.48	107.54	8.35	76.78	84.60	8.46	77.79	57.97

*Note:* The conversion of epoxidation and the viscosity of DESs at different temperature were also listed.

^a^ Epoxy value.

^b^ Conversion of double bonds.

^c^ Viscosity.

At 40°C, a conversion of 66.29% was obtained for ChCl−xylitol and 69.24% for ChCl−urea, but the conversion and epoxy value of these two DESs were significantly reduced. This reduction in conversion and epoxy value can be attributed to the higher viscosity of these two DESs which slowed down the rate of mass transfer. However, the rate of mass transfer was almost unaffected in the ChCl−glycerol system due to its low viscosity, and the conversion using ChCl−glycerol exceeded 70%. As the temperature continued to increase, the conversion of the double bonds did not increase significantly with the decreasing viscosity.

This phenomenon could be attributed to the strong volatility of H_2_O_2_, which even with the decomposition of hydrogen peroxide, resulted in incomplete reaction or other types of reactions. The decrease in viscosity also implied that hydrogen bonding in the catalysts was weakening to a certain extent. We observed that the DESs exhibited excellent catalytic activity at 50°C, with the conversion of epoxidation in all three DESs exceeding 75%, and the epoxy value exceeding 8.0. This result indicates that the DESs system displayed a better preference for the epoxidation under 50°C for 18 h.

### 3.4 Reaction Proportion (DES: Oil)

**FIGURE 4:**
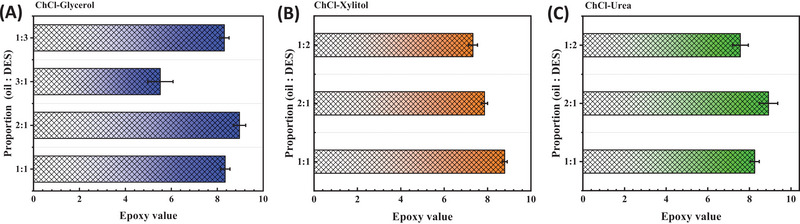
ELO with different proportions of DES; (A) ChCl‐glycerol, (B) ChCl‐xylitol, (C) ChCl‐urea.

After optimizing the reaction conditions, the viscosity of the DESs was found to be crucial for the epoxidation system. The impact on mass transfer during the reaction decreased as the amount of DES added to the reaction system decreased. To study the ratio of vegetable oil to DES, the ELO was conducted at 50°C for 18 h using different ratios of linseed oil to DES (2:1, 1:1, 1:2, and 1:3). The epoxy value of the epoxidation at different ratios was shown in Figure 4A–C. Due to the high viscosity of the ChCl−xylitol and ChCl−urea systems, only three addition ratios (2:1, 1:1, and 1:2) were studied to avoid making the reaction system too viscous and affecting the analysis and processing of samples. The optimal ratio of linseed oil to DES was 1:1 for the ChCl‐xylitol system, resulting in an epoxy value of 8.79 and a double bond conversion of 80.83%. Adding more or less DES caused a significant drop in epoxy value from 8.8 to 7.5. For the ChCl‐urea and ChCl‐glycerol systems, the best ratio of linseed oil to DES was 2:1, resulting in epoxy values of 8.92 and 8.97, respectively, and double bond conversions of over 82%. During the enzymatic epoxidation reaction, high concentrations of hydrogen peroxide could damage enzyme activity, but adding DESs could stabilize enzyme structure [37, 38, 41, 42]. Additionally, because the hydrogen bond network was very strong and not easily destroyed, it could be added to the reaction system as a reaction solvent without affecting the original reaction. The formation of a hydrogen bond network resulted in a certain viscosity, so the amount added played a crucial role in stabilizing enzyme structure and acting as a solvent in the reaction. Thus, the optimal ratio of linseed oil to DES was found to be 2:1 for the ChCl‐urea and ChCl‐glycerol systems, and 1:1 for the ChCl‐xylitol system.

### 3.5 Analysis of Products

TGA: The TG curves of the different samples were presented in Figure 3B. It was observed that all the products displayed three thermal decomposition stages. In the first stage, the inflection point of temperature from ELO catalyzed by Novozym435 using ChCl−xylitol and ChCl−urea was found to be 338.51°C, which was higher than the temperature of 336.11°C observed in ChCl−glycerol. This difference could be attributed to the lower conversion of double bonds in ChCl−xylitol and ChCl−urea. However, the highest decomposition temperature of ELO in ChCl−xylitol, which had a higher purity, was slightly higher at 485.51°C compared to the values of 470.71°C in ChCl−urea and 480.51°C in ChCl−glycerol. Moreover, the rate of weight loss in ChCl−xylitol decreased at the high‐temperature stage, indicating excellent high‐temperature resistance of the product. These results suggest that this product could be a suitable engineering material for industrial applications.

**FIGURE 5:**
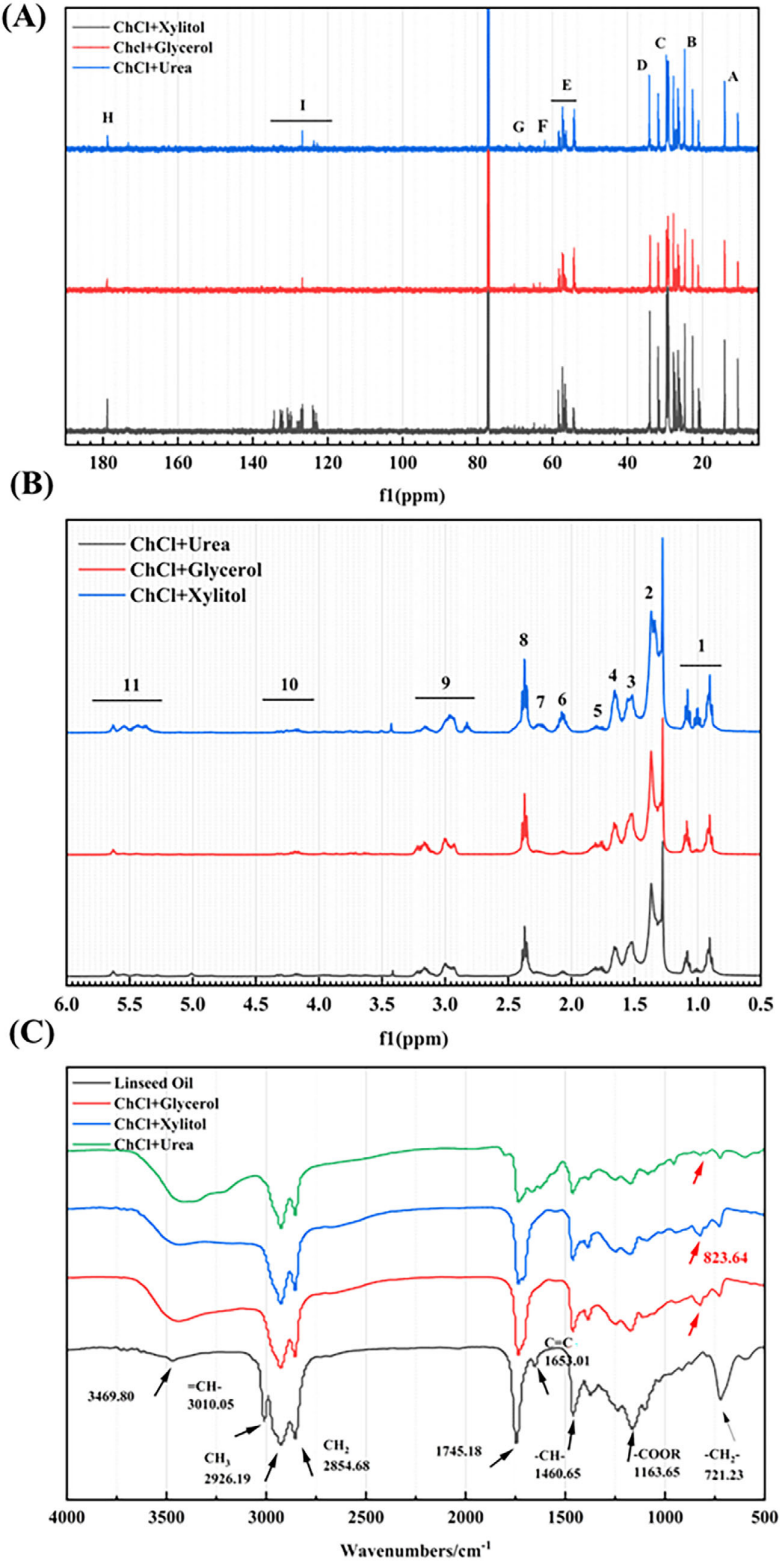
(A) ^13^C NMR spectra of ELO used ChCl−xylitol, ChCl−glycerol, and ChCl−urea under optimal conditions in CDCl_3_; (B) ^1^H NMR spectra of ELO used ChCl−xylitol, ChCl−glycerol, and ChCl−urea under optimal conditions in CDCl_3_; (C) FT‐IR spectra of LO and ELO obtained by three DESs under optimal conditions. The groups associated with wave numbers are highlighted by the dashes.

FT‐IR: Figure 5C shows the FT‐IR spectra of linseed oil and epoxy linseed oil. The absorption peaks were assigned according to literature. The peak at 1163.65 cm^−1^ corresponded to the stretching vibrations of ─COOR from fatty acids in linseed oil. Additionally, the deformation peak of ─CH_2_─ was observed at 721.23 cm^−1^. The peaks at 1460.65 cm^−1^ were formed by the asymmetrical ─C─H deformations in methyl and methylene groups. The absorption peak at 3010.05 cm^−1^ was associated with═C─H─ stretching vibrations. The peak at 1653.01 cm^−1^ resulted from C═C bonds, while the methyl and methylene stretching vibration absorption peaks were observed at 2926.19 and 2854.68 cm^−1^. A strong absorbance band of ─C═O at about 1745.18 cm^−1^ was identified, and the peaks at 3469.80 cm^−1^ were related to ─OH─ vibrations from the raw oil's small amount of free fatty acids. As shown in Figure 5C, all the absorbance peaks associated with linseed oil also appeared in the spectra of the DES‐catalyzed products, except for the asymmetrical stretching vibrations of the epoxy group at 823.64 cm^−1^, indicating that epoxidation reactions had taken place. In general, the spectra of epoxy linseed oil from different DESs had similar absorbance peaks and relative intensities, which suggested that the three DESs could significantly facilitate the chemo‐enzymatic epoxidation reaction of linseed oil.

^1^H NMR: The ^1^H NMR spectral signal peaks of linseed oil were shown in Figure S1, and those of epoxy linseed oil under optimal conditions were numbered from 1 to 11 in Figure 5B. The appearance of large amounts of epoxy groups in epoxy linseed oil was the significant difference from that of linseed oil. The proton peaks at δH (2.85–3.25) were derived from ─CHOCH─ (epoxidation). Furthermore, the peak area at 5.25−5.75 ppm of epoxy linseed oil in the ChCl−glycerol and ChCl−urea systems decreased, indicating a higher conversion of double bonds in the ChCl−glycerol system. In the 1H NMR spectra of the ChCl−xylitol system, many small peaks were observed around the main peaks, and a small peak was also present in the 1H NMR spectra of the ChCl−urea system in Figure 5B. These small peaks were due to side reactions, namely ring opening of epoxidation.

^13^C NMR: The ^13^C spectral signals of the epoxy linseed oil were annotated from A to H (Figure S2). Two clusters of peaks were found at 53.31 and 56.92 ppm (marked with E) in Figure 5A, which indicated the presence of the epoxy product. The ─CH_2_─ internal groups were assigned to carbons with a chemical shift between 22.51 and 32.53 ppm. The carbons (marked with D) at 34.31 ppm were attributed to linked to the ─C═O. The carbon signals at 62.15 and 68.79 ppm were caused by ─CH_2_─ and ─CH─ carbon resonances, respectively, connected to ─CO─ from an ester group. Additionally, signals from 120 to 130 ppm (marked I) were detected in the products, indicating incomplete conversion of double bonds. However, the appearance of strong signals in Figure 5A confirmed that the ChCl−xylitol system possessed a lower conversion than that of ChCl−glycerol and ChCl−urea. Therefore, it could be concluded that the conversion was better with the ChCl−glycerol and ChCl−urea system. The weak signals at δc 64.2 in Figure 5A may correspond to carbons generated in the ring opening polymerization reaction. The spectra suggested that the use of ChCl−xylitol as the solvent under optimal conditions yielded a lower conversion in the epoxidation reaction of linseed oil.

## 4 Conclusion

DESs have the potential to be a sustainable and environmentally friendly alternative to supercritical fluids, ionic liquids, and organic solvents. As solvents for enzymatic epoxidation, they not only protect the structural integrity of biological enzymes from the harmful effects of strong oxidants, but are also cost‐effective, recyclable, and reusable. When the choline chlorine‐glycerol system accounted for half of the raw material, the viscosity of the reaction system was only 91.35 (V.MPA·s), the epoxy value was 8.97, and the double bond conversion rate was 82.48%. The characterization verified the synthesis of the product. The process has laid a solid foundation for enzymatic synthesis of epoxy vegetable oil using DES, and provides a more environmentally friendly, and economical method for the synthesis of various plasticizers in the future. Furthermore, to fully showcase the wide applicability of the system, other factors such as the type of enzyme used in the chemo‐enzymatic epoxidation reaction should also be considered and discussed specifically.

## Conflicts of Interest

The authors have declared no conflicts of interest.

## Supporting information



Supporting Information

## Data Availability

The data that support the findings of this study are available from the corresponding author upon reasonable request.

## References

[1] A. Cherepanova , E. Savel'ev , L. Alieva , I. Kuznetsova , and V. Sapunov , “A New Green Method for the Production Polyvinylchloride Plasticizers From Fatty Acid Methyl Esters of Vegetable Oils,” Journal of the American Oil Chemists' Society 97 (2020): 1265–1272.

[2] Y. Y. Jiang , F. X. Gao , Q. Liu , T. Song , and Y. D. Shen , “Novel Environmentally Sustainable Plasticizers Based on Ricinoleic Acid for Polyvinyl Chloride: Structure and Properties,” New J. Chem. 48 (2024): 4960.

[3] W. Huang , H. Nan , J. Ma , et al., “Synergistic Plasticizing Effect of Bio‑Based Isosorbide Di‑Epoxidized Oleate on Poly(Vinyl Chloride) Resins,” Polym. Bull. 81 (2024): 11671–11691.

[4] D. Garcia‐Garcia , A. Carbonell‐Verdu , M. P. Arrieta , J. Lopez‐Martínez , and M. D. Samper , “Improvement of PLA Film Ductility by Plasticization With Epoxidized Karanja Oil,” Polym. Degrad. Stab. 179 (2020): 109259.

[5] I. Dominguez‐Candela , V. Fombuena , J. M. Ferri , S. C. Cardona , J. Lora , and V. Fombuena , “Dual Plasticizer/Thermal Stabilizer Effect of Epoxidized Chia Seed Oil (*Salvia hispanica* L.) to Improve Ductility and Thermal Properties of Poly(Lactic Acid),” Polymers 13 (2021): 1283. 33920060 10.3390/polym13081283 PMC8071061

[6] R. Saigal , A. B. Chai , N. S. Saad , and C. N. S. Ying , “Potential of Epoxidized Palm Oil as a Green Alternative to Rubber Processing Oils,” J. Phys.: Conf. Ser. 2169 (2022): 012026.

[7] R. L. Quirino , K. Monroe , C. H. Fleischer III , E. Biswas , and M. R. Kessler , “Thermosetting Polymers From Renewable Sources,” Polym. Int. 70 (2020): 167–180.

[8] J. Thomas and R. Patil , “Enabling Green Manufacture of Polymer Products Via Vegetable Oil Epoxides,” Ind. Eng. Chem. Res. 62 (2023): 1725–1735.

[9] C. Di Mauro , S. Malburet , A. Genua , A. Graillot , and A. Mija , “Sustainable Series of New Epoxidized Vegetable Oil‐Based Thermosets With Chemical Recycling Properties,” Biomacromolecules 21 (2020): 3923–3935. 32790997 10.1021/acs.biomac.0c01059

[10] F. Ashine , S. Balakrishnan , Z. Kiflie , and B. Z. Tizazu , “Epoxidation of Argemone Mexicana Oil With Peroxyacetic Acid Formed In Situ Using Sulfated Tin (IV) Oxide Catalyst: Characterization, Kinetic and Thermodynamic Analysis,” Heliyon 9, no. 1 (2023): e12817. 36685436 10.1016/j.heliyon.2023.e12817 PMC9852661

[11] M. d. J. Jalil , A. Hadi , and I. S. Azmi , “Catalytic Epoxidation of Palm Oleic Acid Using in Situ Generated Performic Acid—Optimization and Kinetic Studies,” Mater. Chem. Phys. 270 (2021): 124754.

[12] C. V. Rajput , R. B. Mukherjee , N. V. Sastry , and N. P. Chikhaliya , “Epoxidized Cassia Fistula Seed Oil as Bio‐Based Plasticizer for Poly(Vinyl Chloride) Soft Films,” ACS Appl. Polym. Mater 4, no. 12 (2022): 8926–8941.

[13] E. Santacesaria , R. Turco , V. Russso , R. Tesser , and M. Di Serio , “Soybean Oil Epoxidation: Kinetics of the Epoxide Ring Opening Reactions,” Processes 8 (2020): 1134.

[14] W. Yan , Z. Wang , C. Luo , et al., “Opportunities and Emerging Challenges of the Heterogeneous Metal‐Based Catalysts for Vegetable Oil Epoxidation,” ACS Sustainable Chem. Eng. 10 (2022): 7426–7446.

[15] M. A. Addli , I. S. Azmi , and M. J. Jalil , “In Situ Epoxidation of Castor Oil Via Synergistic Sulfate‐Impregnated ZSM‐5 as Catalyst,” J. Polym. Environ. 32 (2024): 1593–1601.

[16] M. H´ajek , D. Koci´an , and M. Douda , “Statistical Evaluation of the Epoxidation of Esters From Vegetable Oils and Optimization of Reaction Conditions,” Renewable Energy 213 (2023): 157–164.

[17] L. Xu , Q. Zhao , H. Liu , D. Liao , A. Tang , and Y. Liu , “Efficient Epoxidation of (R)‐(+)‐limonene to Limonene Dioxide Through Peracids Generated Using Whole‐Cell Rhizopus Oryzae Lipase,” Bioresour. Technol. 415 (2024): 131645. 39419407 10.1016/j.biortech.2024.131645

[18] A. F. Aguilera , P. Lindroos , J. Rahkila , M. M. Klimov , P. Tolvanen , and T. Salmi , “Lipase Catalyzed Green Epoxidation of Oleic Acid Using Ultrasound as a Process Intensification Method,” Chemical Engineering Process 174 (2022): 108882.

[19] M. Kirpluks , R. Pomilovskis , E. Vanags , A. Abolins , I. Mierina , and A. Fridrihsone , “Influence of Different Synthesis Conditions on the Chemo‐Enzymatic Epoxidation of Tall Oil Fatty Acids,” Process Biochemistry 122 (2022): 38–49.

[20] E. Tohfegar and A. Habibi , “Magnetic Whole‐Cell Biocatalyst Based on Intracellular Lipases of *Candida catenulata* as Promising Technology for Green Synthesis of Epoxy Fatty Acids,” J. Biotechnol. 393 (2024): 117–127. 39098744 10.1016/j.jbiotec.2024.07.019

[21] N. Sarmah , V. Mehtab , K. Borah , A. Palanisamy , R. Parthasarathy , and S. Chenna , “Inverse Design of Chemoenzymatic Epoxidation of Soyabean Oil Through Artificial Intelligence‐Driven Experimental Approach,” Bioresour. Technol. 412 (2024): 131405. 39222857 10.1016/j.biortech.2024.131405

[22] S. Nieto , I. Lozano , F. J. Ruiz , J. F. Costa , R. Villa , and P. Lozano , “Sustainable Synthesis of New Antioxidants From Hydroxytyrosol by Direct Biocatalytic Esterification in Ionic Liquids,” Molecules (Basel, Switzerland) 29 (2024): 5057. 39519698 10.3390/molecules29215057 PMC11547527

[23] A. Prabhune and R. Dey , “Green and Sustainable Solvents of the Future: Deep Eutectic Solvents,” J. Mol. Liq. 379 (2023): 121676.

[24] M. Pätzold , S. Siebenhaller , S. Kara , A. Liese , C. Syldatk , and D. Holtmann , “Deep Eutectic Solvents as Efficient Solvents in Biocatalysis,” Trends Biotechnol. 37, no. 9 (2019): 943–959. 31000203 10.1016/j.tibtech.2019.03.007

[25] F. M. Perna , P. Vitale , and V. Capriati , “Deep Eutectic Solvents and Their Applications as Green Solvents,” Current Opinion in Green and Sustainable Chemistry 21 (2020): 27–33.

[26] F. Liu , L. Chen , K. Yin , T. Fan , and Z. Yan , “Sugars as Hydrogen‐Bond Donors Tune the Phase Behavior in a Novel Liquid–liquid Biphasic System Formed by Hydrophilic Deep Eutectic Solvents and n‐Propanol,” Fluid Phase Equilib. 556 (2022): 113393.

[27] B. Shunde , L. Hanyu , L. Hongxin , and P. Wang , “Integration of Natural Deep‐Eutectic Solvent and Surfactant for Efficient Synthesis of Chiral Aromatic Alcohol Mediated by Cyberlindnera Saturnus Whole Cells,” Biochem. Eng. J. 172 (2021): 108053.

[28] D. ˇSibali´c , A. ˇSali´c , B. Zeli´c , et al., “Synergism of Ionic Liquids and Lipases for Lignocellulosic Biomass Valorization,” Chem. Eng. J. 461 (2023): 142011.

[29] L. Li , X. Wu , Y. Pang , H. Lou , and Z. Li , “In Situ Encapsulation of Cytochrome c Within Covalent Organic Frames Using Deep Eutectic Solvents Under Ambient Conditions,” ACS Appl. Mater. Interfaces 15 (2023): 53871–53880. 37945537 10.1021/acsami.3c14479

[30] S. Liu , Q. Qing , R. I. Foster , et al., “Imine‐Linkage Covalent Organic Framework Synthesis in Deep Eutectic Solvent at Ambient Conditions,” J. Cleaner Prod. 434 (2024): 139970.

[31] R. G. G. Ortizo , V. Sharma , M.‐L. Tsai , et al., “Exploring the Potential of Magnetic Deep Eutectic Solvents and DES‐Functionalized Nanomaterials for Food Analysis: Advancements and Current Trends,” Food Bioscience 61 (2024): 104764.

[32] G. Gang , M. Kalpana , H. Reihaneh , L. Mengchen , and X. Xiao , “Deep Eutectic Solvents‐Based Ionogels With Ultrafast Gelation and High Adhesion in Harsh Environments,” Adv. Funct. Mater. 33 (2023): 2207388. 37090954 10.1002/adfm.202207388 PMC10118073

[33] S. Liu , Q. Zhang , S. Gou , L. Zhang , and Z. Wang , “Esterification of Cellulose Using Carboxylic Acid‐Based Deep Eutectic Solvents to Produce High‐Yield Cellulose Nanofibers,” Carbohydr. Polym. 251 (2021): 117018. 33142579 10.1016/j.carbpol.2020.117018

[34] A. Fernández , M. A. Longo , F. J. Deive , M. S. Álvarez , and A. Rodríguez , “Dual Role of a Natural Deep Eutectic Solvent as Lipase Extractant and Transesterification Enhancer,” J. Cleaner Prod. 346 (2022): 131095.

[35] J. Cao , R. Wu , F. Zhu , Q. Dong , and E. Su , “How to Improve the Efficiency of Biocatalysis in Non‐Aqueous Pure Deep Eutectic Solvents: A Case Study on the Lipase‐Catalyzed Transesterification Reaction,” Biochem. Eng. J. 179 (2022): 108336.

[36] B. Nian , G. Liao , Y. Song , Y. Su , C. Cao , and Y. Liu , “Ionic Hydrogen‐Bonding Interaction Controlled Electrophilicity and Nucleophilicity: Mechanistic Insights Into the Synergistic Catalytic Effect of Lipase and Natural Deep Eutectic Solvents in Amidation Reaction,” J. Catal. 384 (2020): 159–168.

[37] J. Yao , C. Li , L. Xiao , et al., “Influence of Natural Deep Eutectic Solvents on Stability and Structure of Cellulase,” J. Mol. Liq. 346 (2022): 118238.

[38] W. Qian , Z. Min‐Hua , and N. Li , “Enzyme‐Friendly Solvent for One‐Pot Chemobiocatalytic Valorization of Fructose Into Valuable Furanics Via 5‑Hydroxymethylfurfural,” ACS Sustainable Chem. Eng. 12 (2024): 17869–17877.

[39] A. Davide , M. Elia , M. Francesco , S. Nejrotti , and C. Prandi , “Combination of Enzymes and Deep Eutectic Solvents as Powerful Toolbox for Organic Synthesis,” Molecules (Basel, Switzerland) 28 (2023): 516. 36677575 10.3390/molecules28020516 PMC9863131

[40] P. Manuela , M. C. Bubalo , and I. R. Redovniković , “Designing a Biocatalytic Process Involving Deep Eutectic Solvents,” J. Chem. Technol. Biotechnol. 96 (2020): 14–30.

[41] S. M. Taklimi , A. Divsalar , B. Ghalandari , et al., “Effects of Deep Eutectic Solvents on the Activity and Stability of Enzymes,” J. Mol. Liq. 377 (2023): 121562.

[42] J. Cao , R. Wu , F. Zhu , Q. Dong , and E. Su , “Enzymes in Nearly Anhydrous Deep Eutectic Solvents: Insight Into the Biocompatibility and Thermal Stability,” Enzyme Microb. Technol. 157 (2022): 110022. 35276453 10.1016/j.enzmictec.2022.110022

[43] F. Z. I. M. Hassani , S. Amzazi , and I. Lavandera , “The Versatile Applications of DES and Their Influence on Oxidoreductase‐Mediated Transformations,” Molecules (Basel, Switzerland) 24 (2019): 2190. 31212686 10.3390/molecules24112190 PMC6600434

[44] A. Francesca , G. Alessandra , C. Paola , L. Tamborini , and R. Gandolfi , “Continuous‐Flow Stereoselective Reduction of Prochiral Ketones in a Whole Cell Bioreactor With Natural Deep Eutectic Solvents,” Green Chem. 24 (2022): 950–956.

[45] B. B. Hansen , S. Spittle , B. Chen , D. Poe , and Y. Zhang , “Deep Eutectic Solvents: A Review of Fundamentals and Applications,” Chem. Rev. 121 (2021): 1232–1285. 33315380 10.1021/acs.chemrev.0c00385

[46] A. Sobhan , V. Ahirekar , M. Hoff , and K. Muthukumarappan , “Derivation and Characterization of Epoxidized Soybean Oil and Epoxy Resin Film Produced Using a Three Step‐Washing Neutralization Process,” Ind. Crops Prod. 198 (2023): 116675.

[47] R. Turco , R. Tesser , V. Russo , T. Cogliano , M. D. Serio , and E. Santacesaria , “Epoxidation of Linseed Oil by Performic Acid Produced in Situ,” Ind. Eng. Chem. Res. 60 (2021): 16607–16618.

[48] S. Sarmad , Y. Xie , J.‐P. Mikkola , and X. Ji , “Screening of Deep Eutectic Solvents (DESs) as Green CO2 Sorbents: From Solubility to Viscosity†,” New J. Chem. 41 (2017): 290–301.

[49] M. R. G. Klaas and S. Warwel , “Complete and Partial Epoxidation of Plant Oils by Lipase‐Catalyzed Perhydrolysis,” Ind. Crops Prod. 9, no. 2 (1999): 125–132.

