# Supplementary material for: Choline‐Based Deep Eutectic Solvents for Enzymatic Preparation of Epoxy Linseed Oil
Source: Eng Life Sci. 2025 Mar 17;25(3):e70016. doi: 10.1002/elsc.70016 (PMC11913718; doi:10.1002/elsc.70016)
Supplement: Supplementary file 1 — Supporting Information [file ELSC-25-e70016-s001.pdf]

Supporting Information.

Choline-based deep eutectic solvents for enzymatic preparation of epoxy vegetable oil from linseed oil

Hui Zhang [1], *Kai Wang* [1], *Shuai Huang* [1], *Ziheng Cui* [1,*], *Biqiang Chen* [1,*]

[1] College of Life Science and Technology, Beijing University of Chemical Technology

[*] Corresponding author Tel. & Fax: +86-10-64429057
E-mail address: cuileft@gmail.com (Ziheng Cui) and Biqiang Chen (chenbq@mail.buct.edu.cn)

Preparation of the Solvents (DESs)

Choline chloride (ChCl) was mixed with glycerol, ethylene glycol, urea, and urea hydrogen peroxide (UHP) in a molar ratio of 1:2. Choline chloride (ChCl) was mixed with α-glucose in a molar ratio of 5:2. Choline chloride (ChCl) was mixed with D-Sorbitol and xylitol in a molar ratio of 1:1. The two components were stirred and heated in an oil bath at 70 °C until a colorless homogeneous liquid was formed. Once cooled completely, the mixture was placed in a sealed container in a dry place.

Determination of Double Bond Content in Vegetable Oils by Gas Chromatography

To prepare the samples for analysis, they were first converted into fatty acid methyl esters (FAMEs). Specifically, 10 μL of the sample was placed in a 10 mL reaction flask and 2 mL of 0.05 mol/L NaOH-CH_3OH was added. The reaction medium was mixed for 5 minutes at 300 rpm and 70 °C, then cooled to room temperature. Next, 1 mL of boron trifluoride methanol solution was added and mixed again, and then the mixture was cooled to room temperature. After that, 2 mL of n-hexane and saturated sodium chloride solution were added and the mixture was shaken and left to stand. Finally, 2 g of anhydrous sodium sulfate were added to absorb water, and the supernatant was collected as the sample to be tested.

The FAMEs were subjected to analysis using a TRACETM 1300E Gas Chromatography system (Thermo Fisher Scientific, China) equipped with an RTX-Wax capillary column (30 cm × 0.25 mm × 0.25 μm, Agilent). The column was initially set at 180 °C for 1.5 min, followed by an increase to 210 °C at 10 °C/min and maintained at this temperature for 2 min. Subsequently, the temperature was increased to 220 °C at 5 °C/min and held at this temperature for 5 min. The detector was set at 300 °C. An automatic injection of 1 μl of sample was carried out, and each sample was detected for

a duration of 20 min. Component FAME Mix served as the internal standard for the analysis. The determination of unsaturated fatty acids was achieved through External Standard Gas Chromatography.

The vegetable oil was analyzed and the degree of methyl esterification was detected. This allowed for the determination of the relative content of various fatty acids present in the oil, which could then be used to calculate the total number of double bonds in the oil.

Mole number of double bond in 1 g vegetable oil:

$$\frac{(W-92)\times a}{W\times 282}+\frac{(W-92)\times 2b}{W\times 280}+\frac{(W-92)\times 3c}{W\times 278} \quad (1)$$

The molecular weight of the vegetable oil was represented by W, while a, b, and c indicated the content of oleic acid, linoleic acid, and linolenic acid, respectively. The molecular weight of glycerol was 92 g/mol.

Procedure for the Titration Method

The epoxy value is the amount of oxygen present in the ethylene oxide group per 100 g of the sample. The determination of the epoxy value was carried out according to the method described in the literature **错误!未找到引用源。**.

$$EV=\frac{\left[V-\left(V_1-{V_2}/{M_0}\times M_1\right)\right]N\times 0.016}{M_1}\times 100 \quad (2)$$

The epoxy value (EV) of the product was expressed as a percentage (%). To determine the EV, the volume of sodium hydroxide standard solution (V) added to the blank group in milliliters (ml) was subtracted from the volume of sodium hydroxide standard solution (V_1) added to the product in ml. The acid value of the product was determined by using a standard sodium hydroxide solution, with the volume recorded as V_2 in ml. The sodium hydroxide standard solution concentration (N) was measured in moles per liter (mol/L). The mass of the sample (M_0) was recorded in grams (g), while

the mass of the sample used in the acid value analysis (M_1) was also recorded in g. Finally, the molar mass of oxygen (0.016 g/mol) was used in the calculations.

$$EV_{tox} = \frac{M_O}{M_O + \frac{2M_I}{IV_{iod}} \times 100} \tag{3}$$

$$\text{yield } (\%) = \frac{EV}{EV_{tox}} \times 100 \tag{4}$$

Theoretical epoxy value (EV_{tox}) in Eq. (3) referred to the complete conversion of 100 g of linseed oil. The iodine value of linseed oil (IV_{iod}) was determined using the Wijs method [错误!未找到引用源。], and was used in the calculation. M_O and M_I corresponded to the relative atomic mass of oxygen and iodine atoms, respectively.

In Eq. (4), the epoxide yield was calculated based on the expected epoxy value, which was determined to be 10.857 from the iodine value of linseed oil [错误!未找到引用源。]. The expected epoxy value represented the theoretical epoxy value in 100 g of linseed oil.

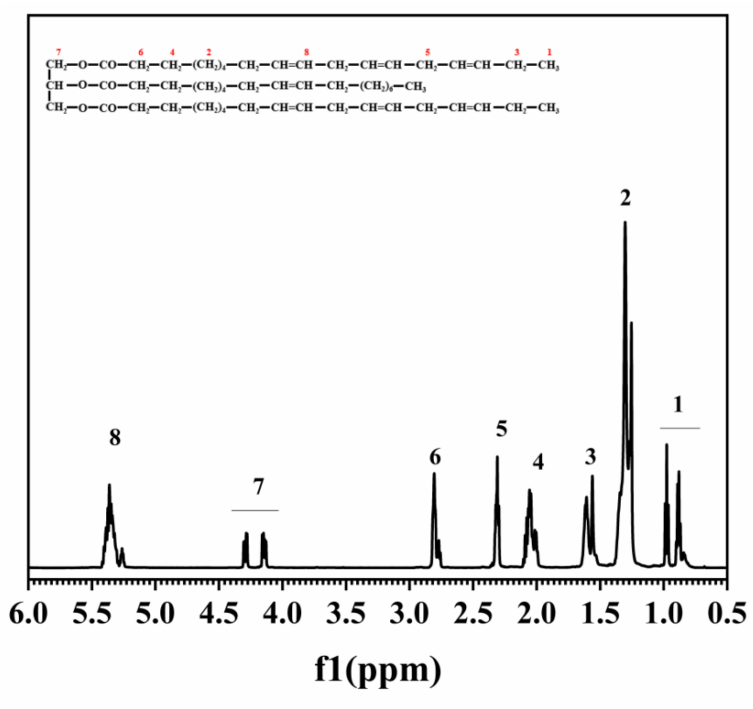

Figure S1. ^{1}H NMR spectra of LO in $CDCl_3$; Hydrogen signals were annotated with different positions from molecular structure in upper left of the figure.

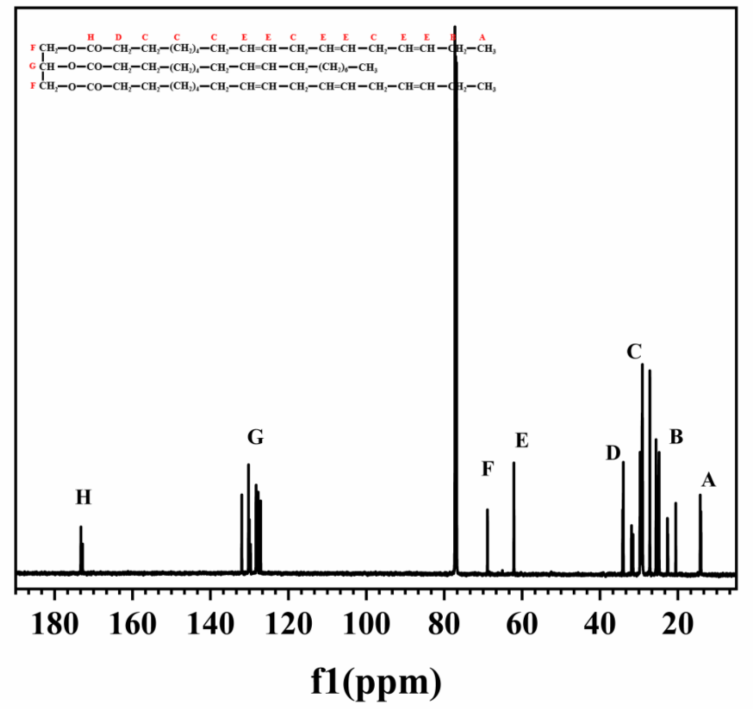

Figure S2. ^{13}C NMR spectra of LO in $CDCl_3$; Carbon signals were annotated with different positions from molecular structure in upper left of the figure.

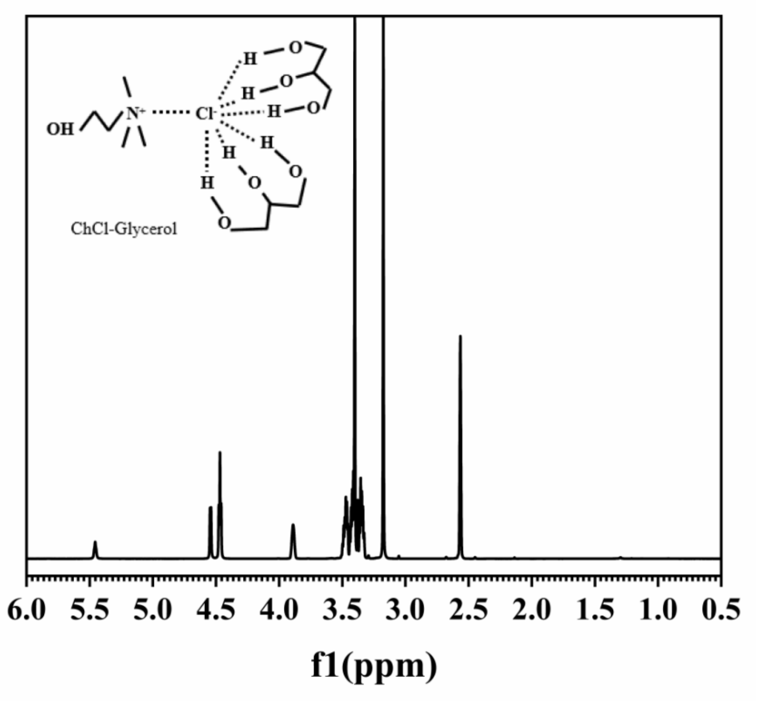

Figure S3. ^{1}H NMR spectra of ChCl-Glycerol in DMSO-d_6.

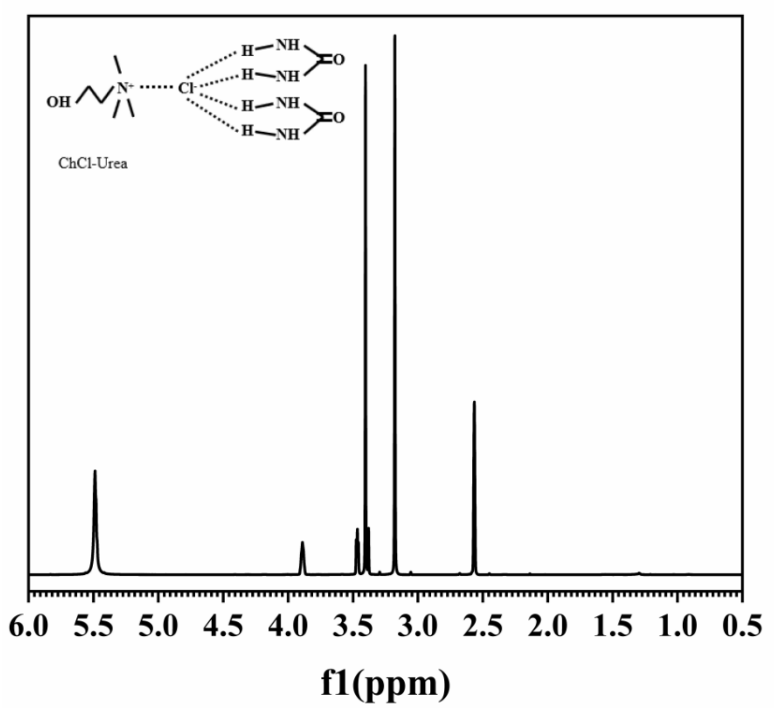

Figure S4. ^{1}H NMR spectra of ChCl-Urea in DMSO-d_6.

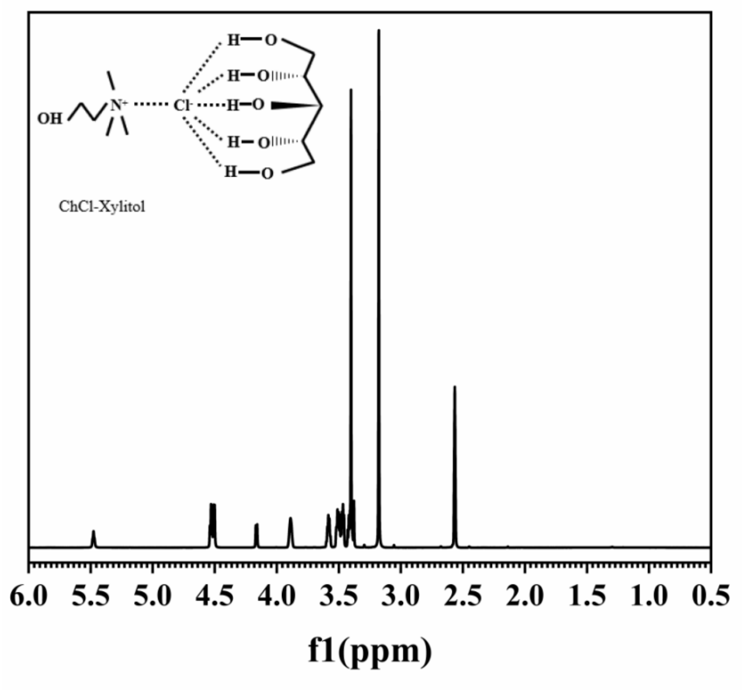

Figure S5. ^{1}H NMR spectra of ChCl-Xylitol in DMSO-d_6.

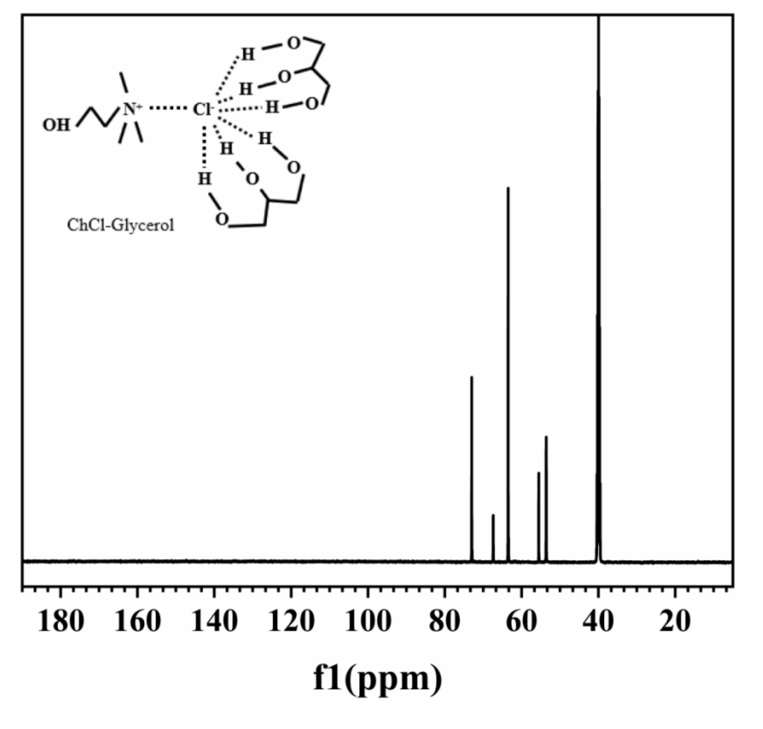

Figure S6. ^{13}C NMR spectra of ChCl-Glycerol in DMSO-d_6.

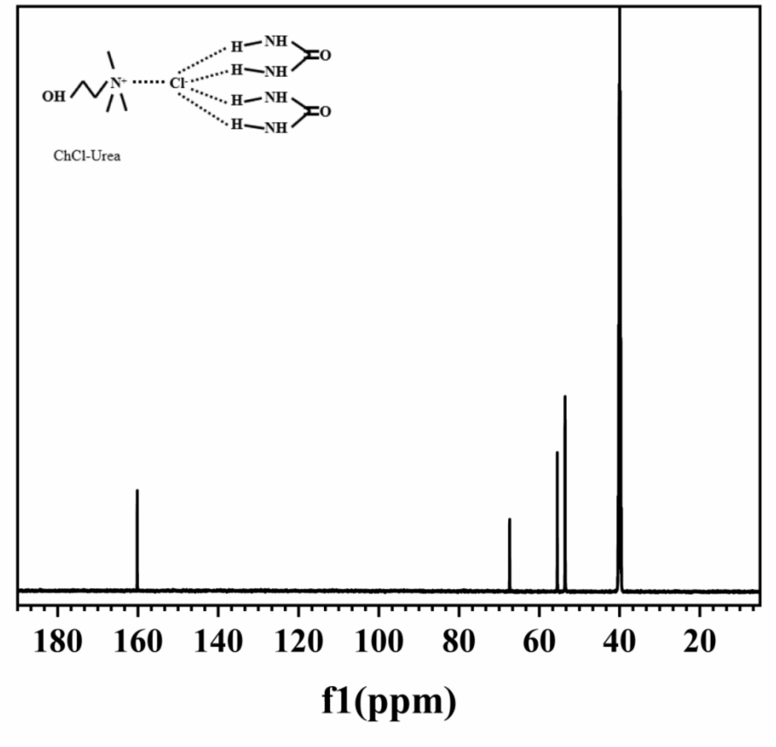

Figure S7. ^{13}C NMR spectra of ChCl-Urea in DMSO-d_6.

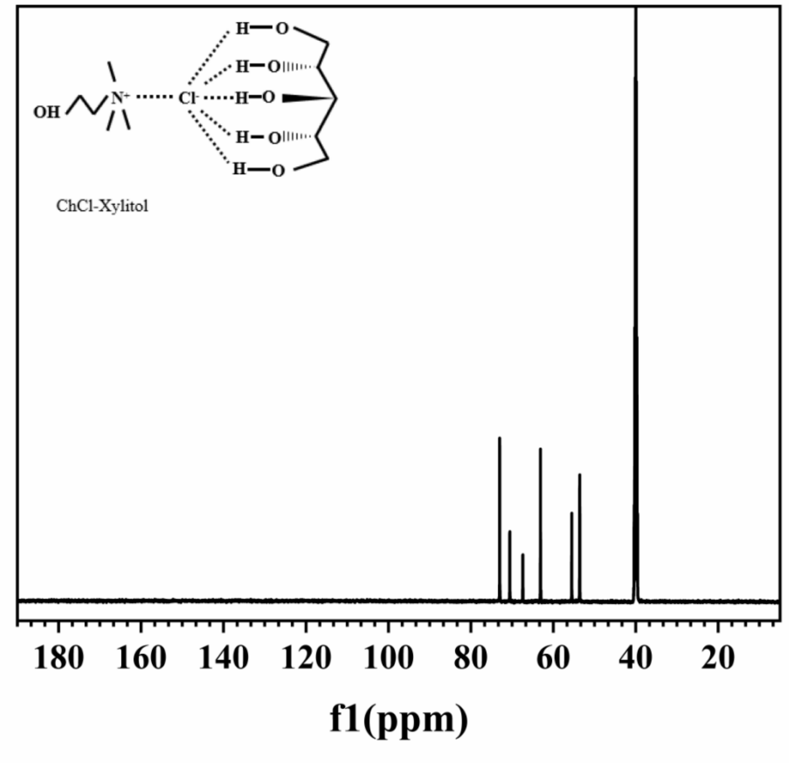

Figure S8. ^{13}C NMR spectra of ChCl-Xylitol in DMSO-d_6.

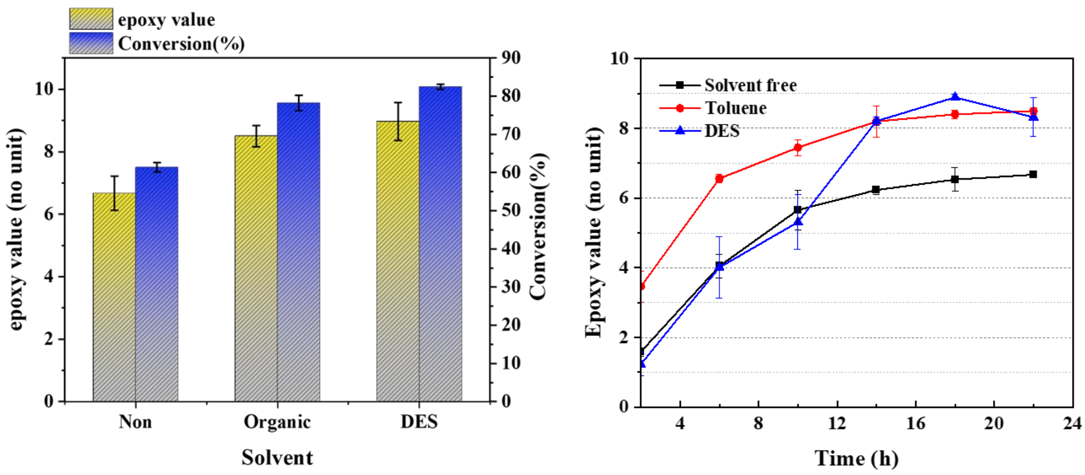

Figure S9. Application of different solvents
